# 3D Polymer Gel Dosimeters with iCBCT 3D Reading and polyGeVero-CT Software Package for Quality Assurance in Radiotherapy

**DOI:** 10.3390/ma17061283

**Published:** 2024-03-11

**Authors:** Marek Kozicki, Piotr Maras, Malwina Jaszczak-Kuligowska

**Affiliations:** 1 Department of Mechanical Engineering, Informatics and Chemistry of Polymer Materials, Faculty of Material Technologies and Textile Design, Lodz University of Technology, Zeromskiego 116, 90-543 Lodz, Poland; malwina.jaszczak@p.lodz.pl; 2 Department of Radiotherapy Planning, Copernicus Hospital, Pabianicka 62, 93-513 Lodz, Poland; piotr.maras@wp.pl

**Keywords:** medical accelerator, radiotherapy dosimetry, 3D dosimetry, gel dosimeter

## Abstract

Dynamically evolving radiotherapy instruments require advancements in compatible 3D dosimetry systems. This paper reports on such tools for the coincidence test of the mechanical and radiation isocenter for a medical accelerator as part of the quality assurance in routine radiotherapy practice. Three-dimensional polymer gel dosimeters were used in combination with 3D reading by iterative cone beam computed tomography and 3D data processing using the polyGeVero-CT software package. Different polymer gel dosimeters were used with the following acronyms: VIP, PAGAT, MAGIC, and NIPAM. The same scheme was used for each dosimeter: (i) irradiation sensitivity test for the iterative cone beam computed tomography reading to determine the appropriate monitor unit for irradiation, and (ii) verification of the chosen irradiation conditions by a star-shot 2D irradiation of each 3D dosimeter in the direction of performing the test. This work concludes with the optimum monitor unit per beam for each selected 3D dosimeter, delivers schemes for quick and easy determination of the radiation isocenter and performing the coincidence test.

## 1. Introduction

Radiation therapy is a technique that uses ionizing radiation in cancer treatment. Thanks to the development of computers and irradiation techniques, even small tumours can be eliminated with high precision using stereotactic body radiation therapy (SBRT) and stereotactic radiosurgery (SRS) [1,2]. Quality assurance (QA) testing is required to ensure treatment excellence [3,4]. One of them is the coincidence of radiation and the mechanical isocenter [5]. The radiation isocenter is defined as the centre of the smallest sphere through which all straight lines passing through the X-ray source and the centre of the collimator of the medical accelerator pass or are tangential [5]. This can be investigated by the following tests: star shot measurements [6,7,8] using 2D films and electronic portal imaging devices (EPIDs) [9,10,11,12,13,14] or the Winston–Lutz test [15]. The outcome of the tests is the size (radius) and distance (offset) to a predetermined point in space relative to the mechanical isocenter. A new approach to determining the isocenter uses the dynamically developing high-resolution 3D radiotherapy dosimetry [16,17]. Therefore, this work is devoted to the study of the conditions for determining the radiation isocenter using a tool consisting of 3D polymer gel dosimeters with different radiation dose sensitivities, iterative cone-beam-computed tomography (iCBCT) reading, and the polyGeVero-CT software package with dedicated algorithms [18].

The main element of 3D radiotherapy dosimetry is the 3D dosimeter. The first proposal was the 3D Fricke gel dosimeter, which was a Fricke dosimeter solution in a physical gel matrix [19]. Irradiation of such a dosimeter converted Fe^2+^ into Fe^3+^ ions, followed in 3D by magnetic resonance imaging (MRI). Subsequent studies concerned both this dosimeter and the proposals of other systems, such as polymer gel dosimeters [20,21,22,23,24,25,26,27,28,29,30,31,32,33,34,35,36], radiochromic gel dosimeters [37,38,39,40,41,42,43,44,45,46], radiofluorogenic gel dosimeters [47,48,49,50], plastic radiochromic dosimeters [51,52,53], lung-mimicking dosimeters [54,55,56], deformable radiochromic dosimeters [57,58,59], or combined dosimeters mimicking, e.g., lungs and muscles in one vial [60]. The response of 3D dosimeters to irradiation is manifested by either radical polymerization and cross-linking of monomeric components (polymer gel dosimeters), formation of coloured products (radiochromic systems), or fluorescence of the formed product after irradiation (radiofluorogenic dosimeters). 

**Figure 1:**
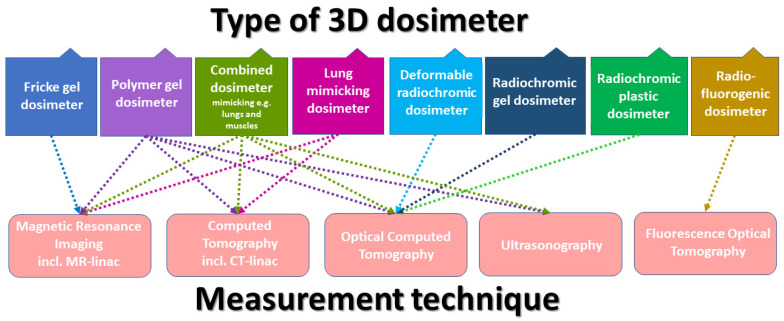
A scheme illustrating the types of 3D dosimeters and their measurement methods.

Alternative 3D reading techniques to the MRI proposed for the first time for Fricke gel dosimeters were also investigated. These are fluorescence tomography to scan only one type of dosimeters (radiofluorogenic) [47], computed tomography (CT) [27,28,61] and optical computed tomography (optical CT or OCT) [62,63] are the most promising, and ultrasonography (USG) [64,65,66] with the lowest applicability so far. Dosimeters can be read using one or more scanning techniques as shown in Figure 1. The data processing challenge for different dosimeters and reading techniques has been reduced by the introduction of dedicated software packages [32,52,54,61]. Three-dimensional dosimetry has been clinically tested [16,17] to demonstrate its great potential, but still awaits widespread introduction into routine practice.

In the case of 3D dosimeter measurements using NMR, MRI, CT, OCT, or USG, they should be moved to another room after irradiation. Currently, however, linear accelerators can also be equipped with MRI- or cone-beam-computed tomography (CBCT or iCBCT) [67,68,69,70,71]. In this way, 3D dosimeters can now be scanned without moving them after irradiation. Therefore, recent studies have shown that the NIPAM 3D dosimeter can be used with CBCT for 3D scanning [72,73], and for the same purpose, a combination of a 3D gel dosimeter with MRI can be used to determine the radiation isocenter using the PAGAT and MAGAT dosimeters using the star-shot irradiation pattern [74,75,76,77]. The KI-PVA radiochromic gel dosimeter combined with the Winston–Lutz test was also used to determine the isocenter [78]. Recent work has demonstrated the use of PABIG^nx^ [18] and VIPAR^nd^ (VIP) [79] for radiation isocenter determination with CBCT and iCBCT reading and data processing using the polyGeVero-CT software package. The main conclusions drawn from [18] for the TrueBeam and Halcyon medical accelerators are as follows: (i) iCBCT is superior to CBCT; scan results for VOI had the lowest standard deviation for the iCBCT reconstruction algorithm; therefore, it is recommended to use it to scan other 3D polymer gel dosimeters; (ii) containers made of poly(methyl methacrylate) with a capacity of ~0.6 and ~1 L (PH-5-DD1, GeVero Co., Lodz, Poland) are suitable for isocenter determination (2D and 3D approach), (iii) fiducial markers significantly affect image noise and artifacts that affect isocenter determination—the best results were obtained for the shielded copper wire of the network cable attached to the containers; (iv) a water phantom is not required to scan the dosimeters and they are irradiated and scanned in one place without moving them to another room; (v) a multi-leaf collimator (MLC) gap should preferably be set to 2 mm, and 10,000 MU should be emitted per beam in star-shot irradiation of PABIG^nx^; both PABIG^nx^ and VIP [79] are medium sensitive dosimeters, so they require this number of MUs per beam that can be delivered at a high dose rate in order to shorten the irradiation time and facilitate isocenter determination; (vi) dosimeters should be scanned before and after irradiation; scanning is performed immediately after irradiation, unlike the accepted 3D polymer gel dosimetry scan after post-effect (approximately 24 h or less); (vii) the best scanning mode is Pelvis mode for TrueBeam and Pelvis or Pelvis Large Fast for the Halcyon accelerator, (viii) the polyGeVero-CT software package proved to be reliable in fast and easy data processing to determine isocenter parameters [18]; it has been shown that image processing can be reduced to calculating average values from up to three scans and subtracting the background from the average scan of the irradiated dosimeter [18]. In turn, the results obtained for the VIP dosimeter [79] indicated the possibility of using this dosimeter made of unpurified *N*-vinylpyrrolidone (as delivered), which reduces the labour intensity and cost of VIPAR family dosimeters (also VIP) preparation [33]. In conclusion, the above irradiation and 3D measurement conditions and data processing should be propagated to other 3D polymer gel dosimeters, such as those with high dose sensitivity, which have not been studied with iCBCT reconstruction. 

The aim of the work is to examine and compare the conditions for the test of coincidence of the mechanical and radiation isocenter for the TrueBeam medical accelerator using 3D polymer gel dosimeters of specific monomer components in the compositions. For this purpose, VIP [30], NIPAM [80], PAGAT [81], and MAGIC [25] were selected. The study design included: (i) irradiation sensitivity test for the iCBCT reading to determine the monitor unit (MU) per radiation beam, and (ii) verification of selected irradiation conditions using star-shot 2D irradiation to perform the coincidence test. This work seeks the optimum monitor unit per beam for each 3D dosimeter, and the irradiation effect adequately visible on iCBCT scans to correctly perform the test of coincidence of the mechanical and radiation isocenter for a medical accelerator. This work also examined the reliability of tools consisting of the 3D polymer gel dosimeters combined with fast 3D iCBCT reading, which takes place without the need to transfer the dosimeters to another computed tomography room, and the polyGeVero-CT software package with dedicated functionalities for data processing of the coincidence test. Some dosimeters, such as NIPAM, MAGIC and PAGAT have never been used in conjunction with iCBCT reading and the software package. The cost and labour related to the application of 3D dosimeters are also discussed. The characterization techniques and instruments used in this study are as follows: (i) irradiation with a medical linear accelerator (TrueBeam, Varian, Palo Alto, CA, USA), (ii) high-resolution 3D reading with iCBCT integrated with the accelerator, and (iii) fast data processing using the polyGeVero-CT software package (GeVero Co., Lodz, Poland).

## 2. Materials and Methods

### 2.1. Preparation of 3D Polymer Gel Dosimeters

In this study, four dosimeters were examined: VIP [30], NIPAM [80], MAGIC [25], and PAGAT [81], each containing different monomers. Apart from these dosimeters, VIPET [82] and PAGAT [83] (higher monomer concentrations with respect to PAGAT reported in [81]) were prepared. However, after preparation, which was performed exactly as reported by other researchers, the dosimeters tightly closed in 1 L poly(methyl methacrylate) containers (PH-5-DD1, GeVero Co., Lodz, Poland) turned white. This opacity was either the effect of precipitation of monomers (*N*,*N′*-methylenebisacrylamide, MBA, in particular) or self-polymerisation. It has been recognized that these dosimeters may not be accurately optimized for high-capacity containers and require further experimentation to obtain a recipe for the preparation of clear and transparent dosimeters. The compositions of the dosimeters examined in this work are presented in Table 1. All dosimeters were prepared as close as possible to the methodology described in the aforementioned works, maintaining the order of adding the ingredients and using a magnetic stirrer with a temperature control system (IKA Works, Staufen im Breisgau, Germany). To prepare the VIP dosimeter, MBA (Sigma Aldrich, Saint Louis, MO, USA) was added first to redistilled water at room temperature. Then, the solution was heated to 50 °C to add gelatine (Sigma Aldrich, Saint Louis, MO, USA). In the next step, the temperature was increased to 55 °C, and after about 20 min of stirring, the solution was cooled to 32 °C to add *N*-vinylpyrrolidone (NVP, freshly distilled, Sigma Aldrich, Saint Louis, MO, USA). Then, oxygen scavengers such as ascorbic acid (AsAc, Chempur, Piekary Śląskie, Poland) and copper(II) sulfate pentahydrate (CuSO_4_ × 5H_2_O, Chempur, Piekary Śląskie, Poland) were added to the solution. To prepare the NIPAM dosimeter, the gelatine was added to water heated to 50 °C and stirred until dissolved. The solution was then cooled to 34 °C for the addition of *N*-isopropylacrylamide (NIPAM, TCI America, Portland, OR, USA) and MBA. Finally, the solution was cooled to 30 °C and tetrakis(hydroxymethyl)phosphonium chloride (THPC, Sigma Aldrich, Saint Louis, MO, USA) was added and stirred for 1 min. 

To prepare the MAGIC dosimeter, gelatine was added to water heated to 50 °C and thoroughly mixed. After the gelatin was completely dissolved, hydroquinone (HQ, Sigma Aldrich, Saint Louis, MO, USA) was added and the solution was cooled to 37 °C to add AsAc and CuSO_4_ × 5H_2_O. Then, methacrylic acid (MMA, Sigma Aldrich, Saint Louis, MO, USA) was added to the solution and stirred until complete dissolution. In this work, the MAGIC dosimeter was prepared several times using freshly purchased methacrylic acid with the addition of 250 ppm MEHQ (methyl hydroquinone ether-stabilizer added by the manufacturer) and another methacrylic acid (over one year old) without information about the addition of MEHQ (both from the same manufacturer and the same number catalog). Preliminary tests were carried out in which MAGIC was produced and irradiated with the content of 250 ppm MEHQ and additionally with and without 0.2% HQ (Table 1). In both cases, the composition was transparent, and no disturbing turbidity was observed after preparation. However, both compositions responded in the same way at the high dose of 50 Gy, showing only slight whitening after dose absorption. In turn, MAGIC prepared using methacrylic acid (no information about the addition of MEHQ was provided) was opaque after preparation but reacted to much smaller doses by significantly whitening the composition. In summary, the MAGIC composition appears to require optimization and detailed study to avoid opacity, and this opaque composition has been used in the current study of coincidence of radiation and mechanical isocenter.

To prepare the PAGAT dosimeter, gelatin was added to heated water (50 °C followed by MBA and left to dissolve. Heating was then turned off to add acrylamide (AA, Sigma Aldrich, Saint Louis, MO, USA) and THPC at 40 °C and 30 °C, respectively. According to the procedures, the VIP dosimeter was prepared by the weight–volume method, and the other dosimeters (NIPAM, MAGIC, and PAGAT) by the weight–weight method. After preparation, all dosimeters were poured into two types of plastic (poly(methyl methacrylate)) containers equipped with pressure-compensating valves: (i) container with dimensions: height 210 mm + filler plug 10 mm, the inner diameter of the main part 90 mm, height of the main part 130 mm, maximum width of the lower lid stand 130 mm, and a volume of approximately 1040 cm^3^ (PH-5-DD1, GeVero Co., Lodz, Poland); (ii) container with the following dimensions: height 170 mm + filler plug 10 mm, inner diameter of the main part 80 mm, height of the main part 90 mm, maximum width of the lower lid stand 120 mm, and a volume of approximately 650 cm^3^ (PH-6-DD2, GeVero Co., Lodz, Poland). Samples were wrapped in aluminium foil to protect them from daylight and allowed to solidify overnight before irradiation. VIP and MAGIC dosimeters were kept at room temperature (about 20 °C), while NIPAM and PAGAT dosimeters were initially cooled in a refrigerator (5 °C) for 2 h and then stored at about 20 °C. For the preparation of all polymer gel dosimeters, the compounds were weighed on a laboratory scale with an accuracy of ±0.1 mg (model: AS220.X2 PLUS, RADWAG, Radom, Poland). Liquid dosimeters were set in volumetric flasks with an accuracy of 0.1–0.4 mL (at 20 °C) for 100–1000 mL flasks (ISO 1042 [84], Boro 3.3, Glassco, Haryana, India), respectively. Liquid compounds were added during preparations of the dosimeters using glass pipettes of 0.03 mL accuracy (AS, ISO 835 [85], LMS, Brigachtal, Germany).

### 2.2. Irradiation of 3D Polymer Gel Dosimeters

One PH-5-DD1 container (GeVero Co., Lodz, Poland) was used for each 3D polymer gel dosimeter to observe the impact of the number of monitor units (MU) on the reading of the polymerized and crosslinked regions using iCBCT (mode Pelvis, mean of 3 series) (TrueBeam, Varian, Palo Alto, CA, USA). The linear accelerator was calibrated according to the IAEA TRS-398 code of practice [86]. The following settings for irradiation were used: 500, 1000, 1500, 2000, 5000, and 10,000 MU, HD MLC (high-definition multi-leaf collimator) gap of 2 mm, 10 MV FFF (flattening-filter-free) photon beam and monitor unit rate of 2400 MU/min. The field set by the MLC jaws was 2 × 3 cm^2^. 

Another PH-5-DD1 container was used for each 3D polymer gel dosimeter to determine the isocenter for the TrueBeam accelerator using the 2D approach [18]. In the top and bottom part of the container, two 2D star-shot irradiations [18] were performed in two selected MU per beam for each 3D polymer gel dosimeter. Each dosimeter was irradiated with four fields; conditions were as follows: 10 MV FFF, 2400 MU/min, 2 mm HD MLC leaf gap, and monitor units per beam: 5000 and 10,000 MU; gantry angles: 0°, 90°, 150° and 240°. The field set by the jaws of MLC was 2 × 3 cm^2^. Markers of the shielded copper wire of the network cable were affixed to the PH-5-DD1 container. The container was placed in the mechanical isocenter of the accelerator using a LAP laser system (accuracy < 1 mm, LAP GmbH Laser Applikationen, Lüneburg, Germany) and the markers were attached to the container.

### 2.3. iCBCT Scanning of 3D Polymer Gel Dosimeters

Each dosimeter was scanned after irradiation with iCBCT (mode: pelvis (standard protocol), mean of 3 series, TrueBeam, Varian, Palo Alto, CA, USA). In this case, they were placed on the accelerator bench and remained in one position for the entire duration of the experiment (exposure and scanning). The scanning parameters of 3D polymer gel dosimeters were established in previous studies [18]. The dosimeters were scanned three times before and after irradiation.

### 2.4. Data Processing

All data presented in this work, the determination of the radiation isocenter, were processed using the polyGeVero-CT software package (v. 1.2, GeVero Co., Lodz, Poland) [18,61]. 

To perform the calculations related to the coincidence test, DICOM images obtained after scanning the dosimeters are imported using the polyGeVero-CT software package, both background images (for the non-irradiated dosimeter, 3 series) and images for the irradiated dosimeter (3 series). The mean series is then calculated for both the background and irradiated dosimeter images. The next step is to subtract the average background series from the average series for the irradiated dosimeter. In the current work, no image filtering was used, which is possible in polyGeVero-CT when image noise reduction is needed before further data processing. The series of star-shot irradiated dosimeters prepared in this way can then be used to calculate the coincidence test. To do this, setting an origin should be done, that corresponds to the mechanical isocenter, using the Set Origin function in the software. The origin is set using fiducial markers that are attached to the containers of the dosimeters [18] and are visible in DICOM images. Afterwards, the Isocenter functionality of the software is launched, which is a pop-up window with advanced tools enabling a coincidence test. The most important are the set of lines that are aligned with the star-shot pattern in the image (in 2D or 3D). Isocenter parameters, location, radius, offset (distance between mechanical and radiation isocenters), and distances of lines to isocenter are calculated automatically. To see the computational process related to the coincidence test, the reader can watch the video on polygevero.com (available, 2 March 2024). 

## 3. Results and Discussion

### 3.1. Impact of MU on the 3D Reading

**Figure 2:**
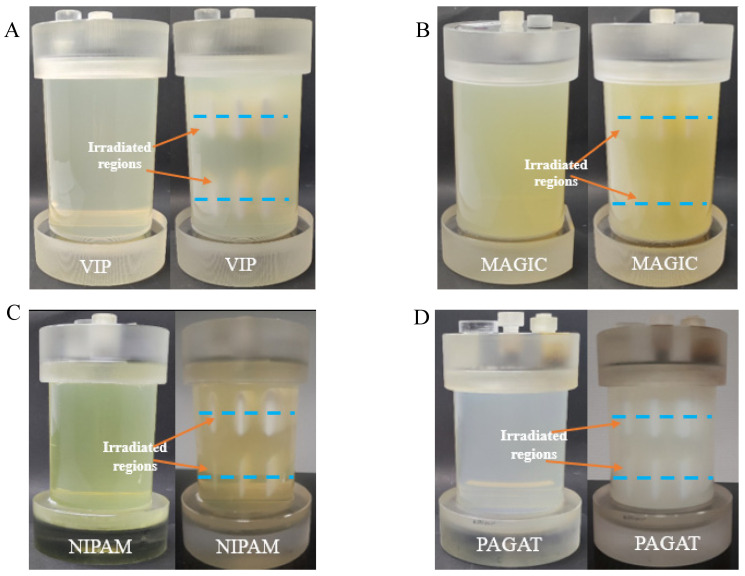
Photographs of non-irradiated and irradiated 3D polymer gel dosimeters. Irradiation was performed to study the impact of the number of monitor units (MU) on the reading of polymerized and crosslinked regions in 3D polymer gel dosimeters using iCBCT (mode pelvis, mean of 3 series) (TrueBeam, Varian, Palo Alto, CA, USA). The dosimeters are in 1 L containers (PH-5-DD1, GeVero Co., Lodz, Poland): VIP (**A**), MAGIC (**B**), and in ~0.6 L containers (PH-6-DD2, GeVero Co., Lodz, Poland): NIPAM (**C**), and PAGAT (**D**). The photographs of the dosimeters are before (left) and after (right) irradiation (500, 1000, and 1500 MU—bottom lines, and 2000, 5000, and 10,000 MU—top lines).

**Figure 3:**
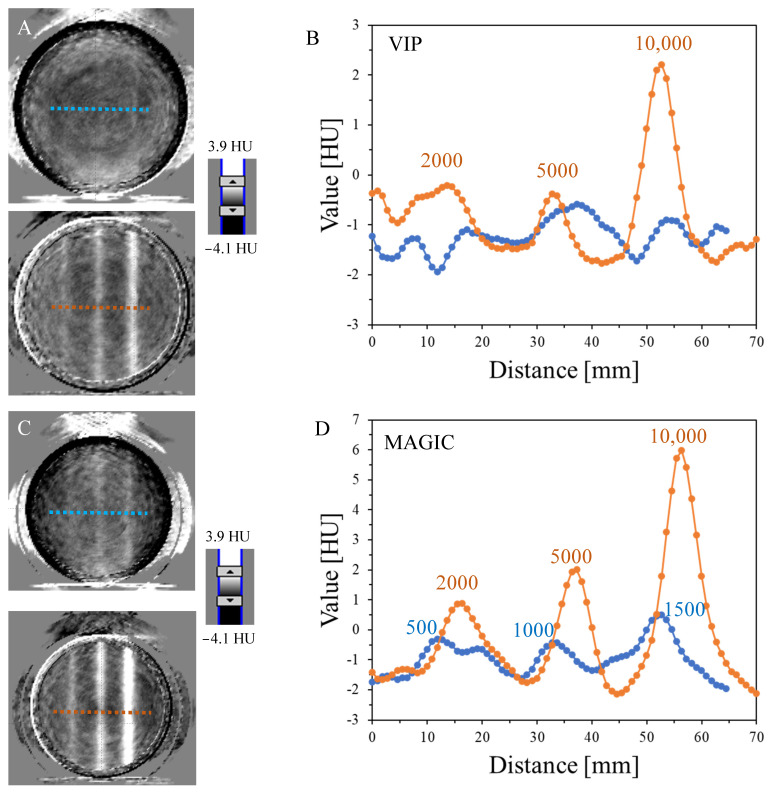

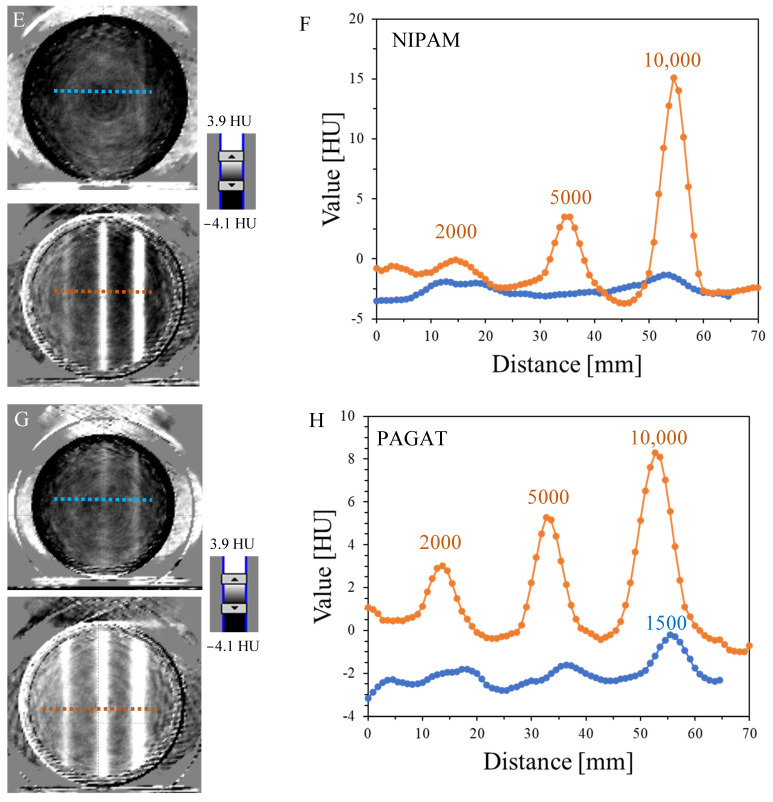
Establishing the radiation field parameters for isocenter determination using 3D polymer gel dosimeters: VIP (**A**,**B**), MAGIC (**C**,**D**), NIPAM (**E**,**F**), PAGAT (**G**,**H**). Both iCBCT transversal images (TrueBeam, Varian, Palo Alto, CA, USA; fixed MLC gap of 2 mm) for two irradiated regions of 500, 1000, and 1500 (first region) and 2000, 5000, and 10,000 MU (second region) (**A**,**C**,**E**,**G**) and profiles (**B**,**D**,**F**,**H**) across these regions are shown. The position where the profiles were taken is indicated on the iCBCT images by blue (first region) and orange (second region) dotted lines. The profiles were smoothed using a mean filter: Kernel mode: 3D, kernel unit: mm, and kernel size 3. Data were processed using the polyGeVero-CT software package (GeVero Co., Lodz, Poland).

VIP, PAGAT, MAGIC and NIPAM 3D polymer gel dosimeters in PH-6-DD1 or PH-6-DD2 containers were used to find one of the radiation field parameters, number of MUs, for the correct and easy determination of the radiation isocenter. Based on the results published elsewhere [18,79], the remaining optimal settings of the TrueBeam accelerator were used, as follows: MLC gap of 2 mm, iCBCT reconstruction algorithm and Pelvis reconstruction mode. Processing of iCBCT data using dedicated polyGeVero-CT algorithms included calculation of mean series from both three scans of background (non-irradiated) and three scans of irradiated samples separately, and mean background series subtraction from the mean series of the irradiated sample [18,79]. The results are presented in Figure 2 and Figure 3. 

The results in Figure 2 show photographs of VIP, MAGIC, NIPAM, and PAGAT dosimeters both before and after irradiation with six bands of different MUs (bottom lines: 500, 1000, and 1500; top lines: 2000, 5000, and 10,000 MU). A very characteristic feature of the polymer gel dosimeters is their transparency after preparation. Visual inspection with the naked eye showed that NIPAM and VIP are relatively transparent compositions, while MAGIC and PAGAT are opaque after preparation and solidification (MAGIC opaquer than PAGAT); all dosimeters are prepared according to original procedures. In consequence, the effect of irradiation is best seen for the transparent dosimeters. The opacity indicates the likely need to optimize a composition and may be a result of both the solubility of the ingredients, the order in which the compounds are added, the quality of methacrylic acid in MAGIC, and the dissolution temperatures during manufacture. In turn, the NIPAM dosimeter seems to be mechanically weak. Particularly noticeable after preparation is that the dosimeter hardly solidifies at room temperature and takes a long time (more than a day) to transform into a solid physical gel. Also, after irradiation, the irradiated areas are fixed in the space of the dosimeter, but they easily fall into resonance when the dosimeter is shaken slightly. This suggests that the composition of this dosimeter could be improved, and the gelatine concentration may need to be increased. The results for the irradiated polymer gel dosimeters showed that they respond to all delivered MUs (Figure 2).

The results in Figure 3 show two transversal images (2D graphs of HU values distribution) passing through two irradiated regions of the dosimeters with six zones irradiated to different MUs and profiles across these regions. The radiodensity of VIP, MAGIC, NIPAM, and PAGAT before irradiation (calculated as mean values from three series for VOIs of 20 × 60 mm^2^), is 18.2 ± 5.41, 23.2 ± 3.56, −3.2 ± 2.14, and 7.7 ± 3.8 HU, respectively. This denotes that MAGIC has the highest radiodensity and NIPAM the lowest. Even though for all polymer gel dosimeters studied, six irradiated regions were visible after irradiation, iCBCT scanning revealed only some of them related to the dosimeter sensitivity to irradiation. Thus, for VIP (Figure 3A,B), bands of 2000, 5000, and 10,000 MU are seen on the corresponding profile; however, the zone of 10,000 MU is clearly visible, and this monitor unit per beam should be first considered for the determination of the radiation isocenter with VIP dosimeter (see Section 3.2 for further discussion). In turn, for the MAGIC dosimeter (Figure 3C,D) all zones but for 500 MU are visible after scanning and data processing. In this case, 5000 or 10,000 MU (or a MU in this range) per beam can be used to determine the radiation isocenter. For the NIPAM (Figure 3E,F) dosimeter, zones apart from 500–2000 give a relatively strong signal in iCBCT scans, and 5000 and 10,000 MU per beam can be used for the determination of the radiation isocenter. This is similar for PAGAT (Figure 3G,H), although 2000 MU per beam can also be considered for this dosimeter due to a relatively strong signal. It should also be noted that a MU between those selected can be considered as an alternative in determining the isocenter. A comparison of the estimated signals recorded by the dosimeters for the MU range examined is shown in Table 2. For comparison, the signals for the VIP and PABIG^nx^ dosimeters obtained in other studies are also presented [18,79]. The results for the VIP dosimeter [18] are close to those obtained in the current study, which denotes reproducibility of the procedure related to the preparation of the dosimeter, irradiation, scanning and data processing. The results for PABIG^nx^ indicate the possibility of using 5000 and 10,000 MU per beam when determining the radiation isocenter.

### 3.2. Radiation Isocenter Determination

**Figure 4:**
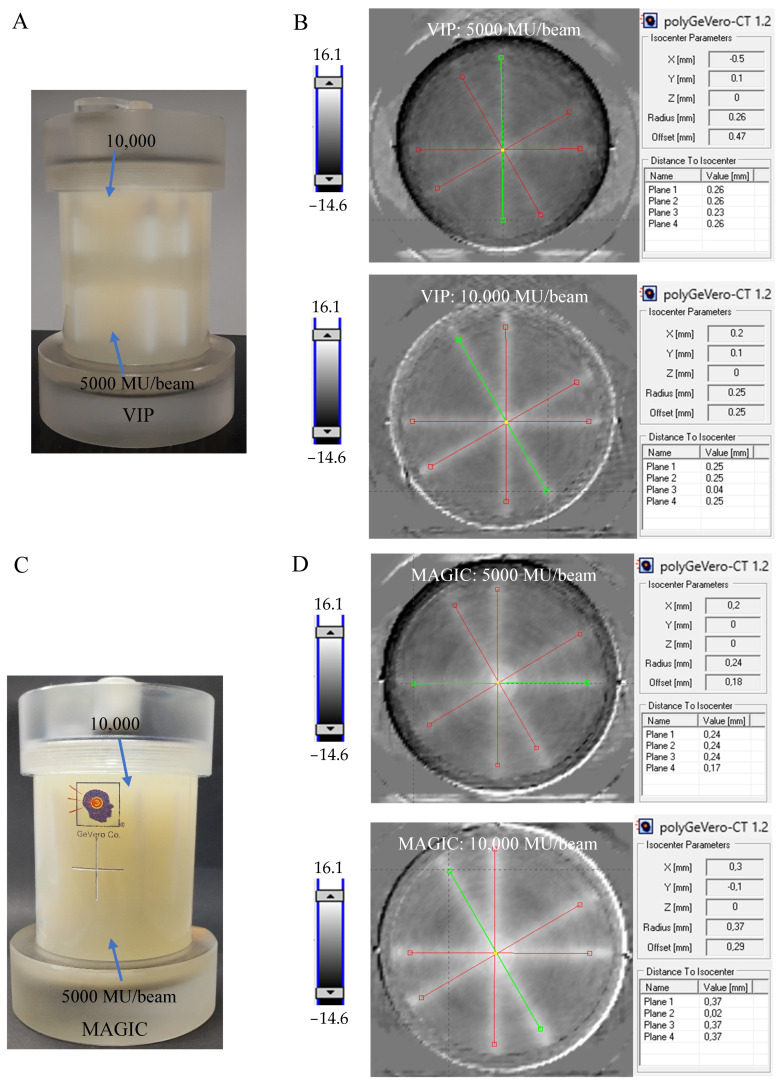

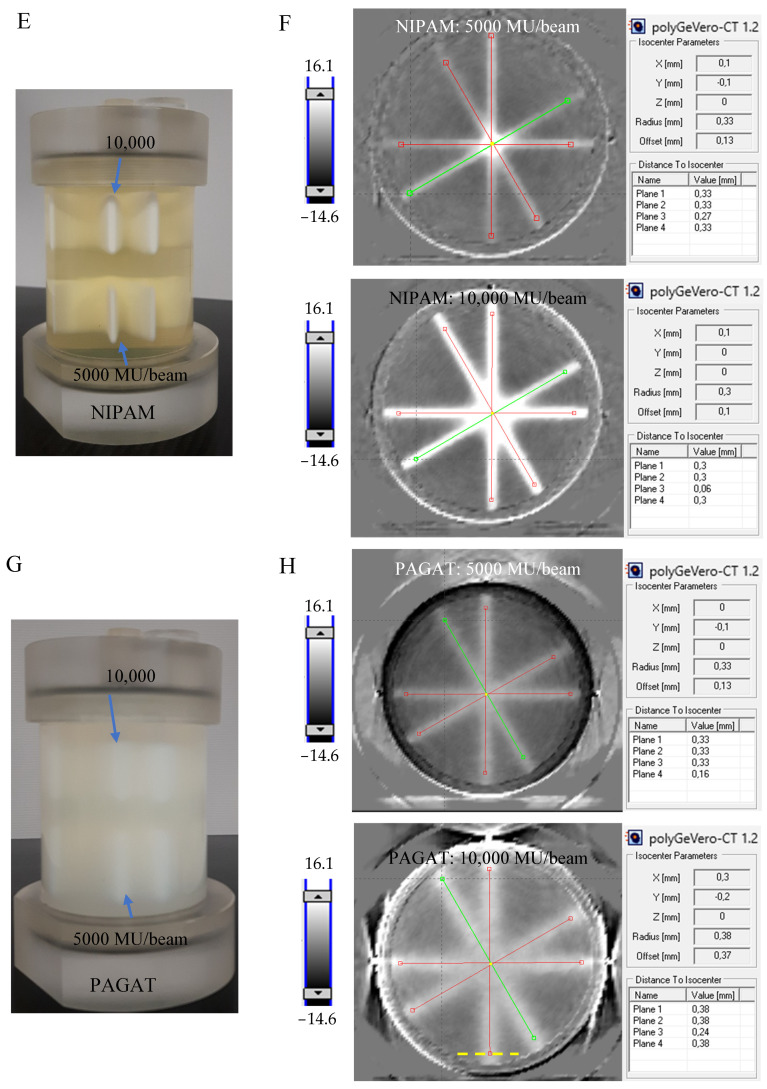
Determination of the radiation isocenter for the TrueBeam accelerator (Varian, Palo, Alto, CA, USA) using 3D polymer gel dosimeters: VIP (**A**,**B**), MAGIC (**C**,**D**), NIPAM (**E**,**F**), and PAGAT (**G**,**H**) and the polyGeVero-CT software package (GeVero Co., Lodz, Poland). Dosimeters in PH6-DD2 containers (~0.6 L, GeVero Co., Lodz, Poland) were irradiated in two regions with the photon beams crossed in a star-shot pattern to investigate the determination of the isocenter for the lower (bottom) and higher (top) monitor units per beam of 5000 and 10,000 (**A**,**B**), 5000 and 10,000 (**C**,**D**), 5000 and 10,000 (**E**,**F**), and 5000 and 10,000 MU (**G**,**H**). In (**A**,**C**,**E**,**G**) are photographs of the dosimeters after irradiation and in (**B**,**D**,**F**,**H**) are the results of data processing with the parameters of the radiation isocenter. Scale bars correspond to HU values.

**Figure 5:**
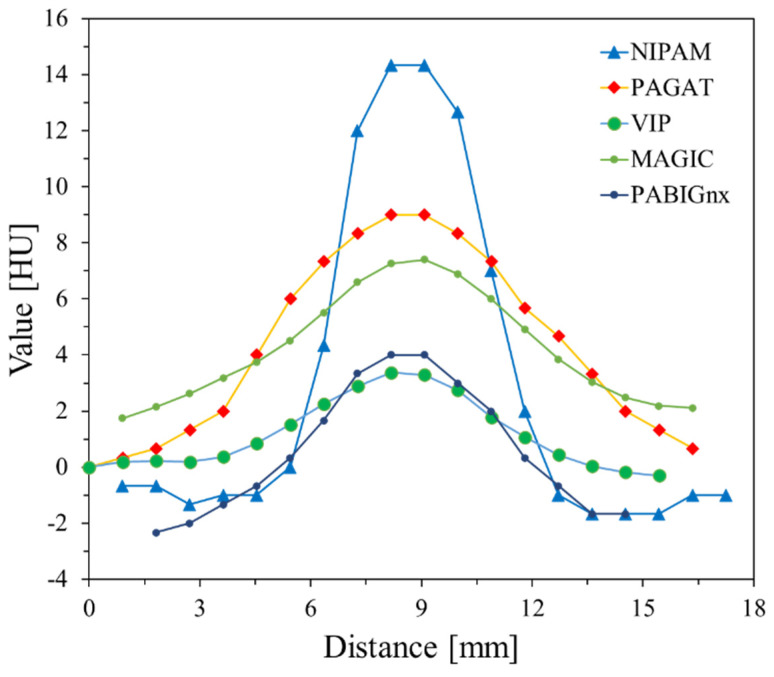
Comparison of profiles for VIP, MAGIC, NIPAM, PAGAT, and PABIG^nx^ from another study for comparison [18]. Profiles drawn for each dosimeter in the position indicated in Figure 3H with yellow dashed line for the higher MU (10,000 MU) per beam used to irradiate the dosimeters (profiles filtered: mean filter, Kernel = 1, mode: 2D, unit: mm).

The results of the test of coincidence of the mechanical and radiation isocenter are presented in Figure 4 for VIP, MAGIC, NIPAM and PAGAT dosimeters. Photographs of each dosimeter are shown (Figure 4A,C,E,G) after irradiation according to the 2D star-shot pattern, where the lower part of each dosimeter was irradiated with a lower MU and the upper part with a higher MU per beam. In turn, Figure 4B,D,F,H contains the results of calculations in the polyGeVero-CT software package, 2D graphs of HU values distribution, which are set to the same HU scale to facilitate comparisons, and the results of the radiation isocenter radius, offset–distance from the mechanical isocenter, and distances of each line (green and red lines in the graphs) to the radiation isocenter. The chemical compositions of each dosimeter allow to compare the effects of irradiation with the naked eye. White zones corresponding to the polymerized monomers and crosslinked polymer are clearly visible for all dosimeters, but in particular for the NIPAM dosimeter. The 2D HU distribution graphs present a distinct star-shot pattern of irradiation. As expected, it is better recognized at higher MU applied. The comparison of these graphs leads to the following conclusions: (i) the monitor unit per beam for the VIP dosimeter should not be lower than 5000 MU, as this would cause problems with the calculations of the radiation isocenter; radiation isocenter calculations for 5000 MU per beam were somewhat difficult due to the relatively low signal for the irradiated areas; thus it may be 5000 MU or more and need not be as much as 10,000 MU. However, such a large monitor unit per beam makes it easier for the user to process data; (ii) in the case of the MAGIC dosimeter, 5000 MU per beam was enough to determine the radiation isocenter; however, it is clear that the composition of the dosimeter should be investigated in more detail in the future due to the high compositional opacity encountered; this means that the monitor unit per beam may be lower for the optimized composition. (iii) Similar conclusions as for the VIP dosimeter apply to the NIPAM dosimeter; additionally, a significant decrease in the recorded HU values for this dosimeter was observed when the monitor unit per beam changes from 10,000 MU to 5000 MU and further to 2000 MU (Table 2). Thus, it seems that decreasing the monitor unit per beam below 5000 MU may be risky for the correct calculations of the radiation isocenter. (iv) A similar conclusion is found for the PAGAT dosimeter—the monitor unit per beam can be between 5000 and 10,000 MU; additionally, as indicated in the previous section, it seems that for this dosimeter the monitor unit per beam may even be much lower than 5000 MU. In addition, it seems that it should not exceed 10,000 MU because the effects of irradiation would result in a significant expansion of the irradiation areas. Comparison of the profiles across the irradiation zones in dosimeters for the selected beam is presented in Figure 5. The sharp one for the NIPAM attracted attention. It is speculated that this dosimeter does not seem to record the effects of irradiation well in penumbra regions unlike other dosimeters. However, this does not affect the calculation of the radiation isocenter using dedicated polyGeVero-CT tools. Too-wide profiles—such as for PAGAT at 10,000 MU per beam, which suggests good recording of irradiation effects for the penumbra region—do not increase the ease of calculations of the radiation isocenter using the mentioned tools.

In summary, each of the dosimeters used in the work proved its usefulness in determining the radiation isocenter. The obtained results (Figure 4B,D,F,H) are similar and fall within the tolerance limits for the star shot measurement; ±1 mm, according to the Task Group 142 report [4].

### 3.3. Workload and Cost of Dosimeters

The workload and time required to perform the coincidence test using VIP, NIPAM, MAGIC, and PAGAT is the sum of operations related to the preparation of dosimeters in dedicated containers, irradiation with a star-shot pattern, scanning using iCBCT and data processing with the polyGeVero-CT software package. The preparation of these dosimeters is similar in terms of labour and time; it takes about 2 h (1 L container). This time may vary when the cooling of the dosimeter composition is required to add some ingredients. However, once prepared, the dosimeter should be kept at room temperature for some time to solidify before irradiation. For those dosimeters that are gelatine-based compositions, it should be about 20 h. However, it has been noted that the NIPAM dosimeter does not solidify at room temperature within 20 h and needs to be cooled for several hours in a refrigerator (e.g., 2 h), after which it can be stored at a temperature of about 20 °C for at least 18 h. If NIPAM is stored after irradiation for several hours at room temperature, it can melt quite easily. In this work, the NIPAM dosimeter was prepared twice. After the first preparation, it was kept at room temperature for about 24 h before irradiation. The dosimeter solidified, but it was very weak in terms of mechanical stability. Moreover, it was unresponsive to 500–2000 MU irradiation. During the second preparation, it was kept at lower temperatures that made it more mechanically robust and responsive to all MU applied. Therefore, it is concluded that NIPAM is not optimized for gelatine concentration and requires further experimentation in this direction. Moreover, after the experiments performed, it was concluded that MAGIC and PAGAT suffer from significant opacity and therefore neither the composition nor the manufacturing procedures nor both were optimized despite their good irradiation-response characteristics. The time needed to irradiate one dosimeter according to the 2D star-shot pattern is about 5–6 min per beam (including settings) for the TrueBeam accelerator (10 MV, FFF, 10,000 MU, 2400 MU/min); ~24 min total. For some dosimeters (MAGIC, PAGAT, NIPAM), the monitor unit per beam can be reduced; in this way, the exposure time is shortened to ~12 min. After irradiation, scanning is performed without transferring/moving the dosimeter. One series is obtained within ~2 min; consequently, three series, like in this work, are obtained within ~6 min. DICOM data processing using the polyGeVero-CT software package takes less than 15 min (all operations in the direction of isocenter parameters, saving the results and printing the report). In summary, to perform the determination of the radiation isocenter with one of the dosimeters, the user needs a total of about 24 h to prepare the dosimeter and less than 45 min for exposure, scanning and data processing. If a 3D dosimeter is purchased from a vendor, this time is shortened to less than 45 min.

The costs of dosimeters estimated on the basis of the actual costs of purchasing components in Poland (June 2023) are specific to a particular dosimeter (Table 3). The highest cost is for NIPAM, and the lowest is for PAGAT. The cost of MAGIC, PAGAT, and PABIG^nx^ (from the former study [18]) is comparable. The cost of VIP is slightly higher than MAGIC, PAGAT, and PABIG^nx^. The costs shown in Table 3 are for a 1 L dosimeter (for PH5-DD1 container, GeVero Co., Lodz, Poland); however, they can be reduced by about 40% for the ~0.6 L dosimeter (for PH6-DD2 container, GeVero Co., Lodz, Poland). The cost of approximately 1 L of NIPAM solution has been estimated elsewhere [72] to be approximately USD 85 (in the US in 2020). Costs may vary in other countries and do not include laboratory labour, electricity consumption, tap and redistilled water consumption, depreciation of equipment, container, packaging and shipping if the dosimeters were intended for shipment.

### 3.4. Uncertainty Budget

The uncertainty budget in 3D radiotherapy dosimetry with the use of 3D dosimeters is dependent on the following applications: measurement of dose distribution or testing of irradiation devices and has been discussed in detail elsewhere [79]. In the current work, it is related to the tests of the irradiation device-the test of the coincidence of the mechanical and radiation isocentres of a medical accelerator. In brief, positioning of the 3D dosimeter during irradiation, spatial registration and image reconstruction, setting the origin in the polyGeVero-CT software package before calculation of the radiation isocenter, alignment of lines/planes during radiation isocenter calculation in polyGeVero-CT, and accuracy of calculation in polyGeVero-CT impact on the uncertainty budget. Consequently, the standard uncertainty [87,88] for determining the radiation isocenter is 1.3 mm regardless of the type of 3D polymer gel dosimeter used in this study.

## 4. Conclusions

Four different 3D polymer gel dosimeters VIP, MAGIC, NIPAM, and PAGAT were tested in the work to determine the radiation isocenter of a medical accelerator (coincidence test of the mechanical and radiation isocenter), which is a periodically necessary test for all devices of this type used in radiotherapy. All dosimeters turned out to be suitable for such tests. However, we noticed that the NIPAM dosimeter is not stable at room temperature, and the MAGIC dosimeter was difficult to use due to the specific and bothersome odour of methacrylic acid; also, this dosimeter affected the containers which became opaque due to corrosion effect.

All studies of determining the radiation isocenter performed in this work with these dosimeters gave results within the tolerance level for the star shot measurement. In addition, this work searched for the parameters of the monitor unit per beam for those dosimeters that are suitable for easy calculation of the radiation isocenter using dedicated tools of the polyGeVero-CT software package. For all dosimeters, the monitor unit per beam can be between 5000 and 10,000 MU maximum. For the VIP dosimeter it should be above 5000 MU and may be lower than 10,000 MU. For MAGIC, NIPAM, and PAGAT, 5000 MU is a sufficient monitor unit per beam. Reducing the monitor unit per beam shortens the total exposure time of the dosimeter. This work also proves that the studied 3D polymer gel dosimeters (MAGIC, NIPAM, and PAGAT first time examined) combined with high-speed iCBCT and the polyGeVero-CT software package are robust tools to quickly perform a coincidence test without moving the post-irradiation dosimeters to another CT room because iCBCT is integrated into the linear accelerator.

## Figures and Tables

**Table 1:** Chemical composition of dosimeters.

Dosimeters Components	VIP (*w*/*v*) [30]	NIPAM (*w*/*w*) [80]	MAGIC (*w*/*w*) [25]	PAGAT (*w*/*w*) [81]
NVP	8%	–	–	–
NIPAM	–	15%	–	–
MMA	–	–	9%	–
AA	–	–	–	3%
MBA	4%	4.5%	–	3%
Gelatine	7.5%	5%	8%	5%
HQ	–	–	0.2%	–
THPC	–	5 mM	–	10 mM
AsAc	0.007%	–	0.0352%	–
CuSO_4_ × 5H_2_O	0.0008%	–	0.002%	–

**Table 2:** Maximal, estimated signal values (HU) for polymer gel dosimeters irradiated in the range of 500–10,000 MU, which were extracted from Figure 3.

Dosimeter	Irradiation (MU)					
	10,000	5000	2000	1500	1000	500
	Signal values (HU)					
VIP	3.9	1.0	-	-	-	-
MAGIC	8.1	3.8	2.1	1.9	1.1	<1.0
NIPAM	18.7	5.9	0.9	-	-	-
PAGAT	8.5	5.6	2.5	2.0	-	-
PABIG^nx^ *	5.8	3.9	1.8	-	-	-
VIP **	4.3	2.1	-	-	-	-

* Based on data published in [18]; ** based on data published in [79].

**Table 3:** Cost of dosimetry solutions (June 2023, Poland).

Dosimeter	The Cost of 1 Litre of Dosimeter (USD)
VIP	75
NIPAM	124
MAGIC	49
PAGAT	42
PABIG^nx^ [18]	57

## Data Availability

The data supporting the reported results are not stored in any publicly archived datasets. The readers can contact the corresponding author for any further clarification of the results obtained.
